# Paradoxical effects of osteoprotegerin on vascular function: inhibiting inflammation while promoting oxidative stress?

**DOI:** 10.1042/CS20211096

**Published:** 2022-03-11

**Authors:** Nhat-Tu Le, Elizabeth A. Olmsted-Davis, Jun-ichi Abe

**Affiliations:** 1 Academic Institute, Department of Cardiovascular Sciences, Center for Cardiovascular Sciences, Houston Methodist Research Institute, Weill Cornell Medical College, Houston, TX, U.S.A.; 2 Department of Cardiology, The University of Texas MD Anderson Cancer Center, Houston, TX, U.S.A.

**Keywords:** NADPH oxidase, osteoprotegerin, RANKL, syndecans, TRAIL

## Abstract

Osteoprotegerin (OPG), also known as osteoclastogenesis inhibitory factor or tumor necrosis factor receptor superfamily member 11B, is well known as a modulator of bone remodeling. The contribution of OPG to cardiovascular disease (CVD) has been suggested, but its molecular mechanism is complex and remains unclear. In the present study, Alves-Lopes et al. (*Clin. Sci. (Lond.)* (2021) **135**(20): https://doi.org/10.1042/CS20210643) reported the critical role of syndecan-1 (SDC-1, also known as CD138), a surface protein part of the endothelial glycocalyx, in OPG-induced vascular dysfunction. The authors found that in endothelial cells (ECs), through SDC-1, OPG increased eNOS Thr^495^ phosphorylation, thereby inhibiting eNOS activity. Furthermore, the OPG–SDC-1 interaction increased reactive oxygen species (ROS) production through NOX1/4 activation. Both the reduced eNOS activity and induced ROS production inhibited NO production and impaired EC function. In vascular smooth muscle cells (VSMCs), the OPG–SDC-1 interaction increased ROS production through NOX1/4 activation, subsequently increased MLC phosphorylation-mediated Rho kinase-MYPT1 regulation, leading to increased vascular contraction. Ultilizing wire myography and mechanistic studies, the authors nicely provide the evidence that SDC-1 plays a crucial role in OPG-induced vascular dysfunction. As we mentioned above, the molecular mechanism and roles of OPG in cardiovascular system are complex and somewhat confusing. In this commentary, we briefly summarize the OPG-mediated signaling pathways in cardiovascular system.

## The OPG–RANKL interaction inhibits vascular inflammation

**Figure 1:**
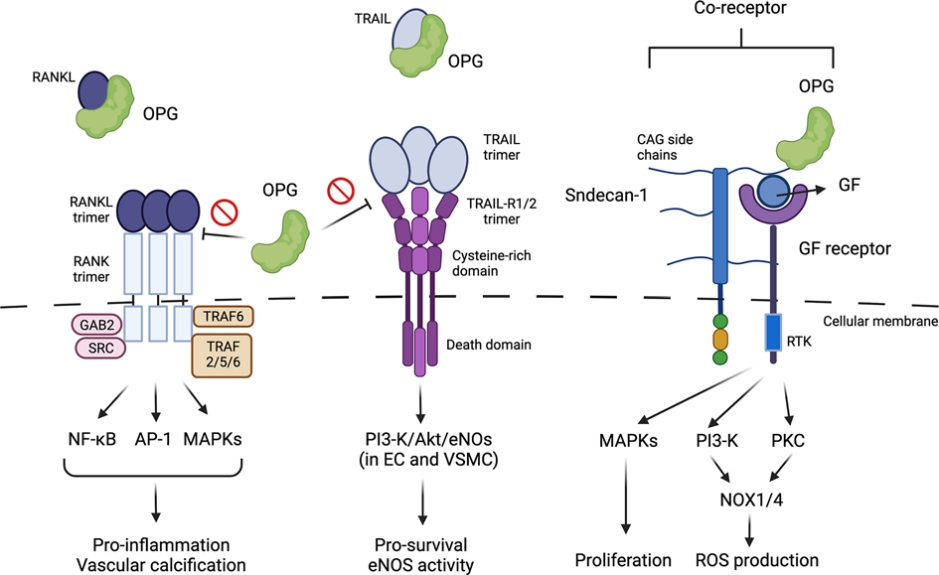
OPG-mediated signaling pathways OPG inhibits RANKL and TRAIL binding to their receptors. In contrast, OPG associates with SDC-1, which promotes growth factor receptor-mediated signaling. RANK itself does not have any kinase activity, but rather signals through binding TRAFs, GRB-associated-binding protein 2 (GAB2), and Src kinase, which assist in initiating the signaling cascade leading to NF-kB, MAPK, and AP-1 activation inflammation, and vascular calcification [5,6]. TRAIL-R1/2 contains a death domain in its C-terminal, and when bound can produce apoptotic signals. However, in endothelial cells (ECs) and VSMCs, TRAIL-R1/2 can increase PI3-K/Akt signaling instead of Fas-associated protein with death domain (FADD)-mediated caspase 8 activation, to promote cellular survival and eNOS activation as described in the studies by Alves-Lopes et al. []. This phenomenon is also reported in cancer cells, and may be one of the mechanisms to explain the resistance to TRAIL-induced cancer cell apoptosis. However, how TRAIL-R1/2 can change its signaling from apoptosis to survival remains unclear [7]. Lastly, OPG associates with SDC-1 at a CAG side chain. CAG side chain contains heparan sulfate (HS), which forms a complex with growth receptor/ligands and acts as a co-receptor. In this manuscript, Alves-Lopes et al. [] showed that OPG can induce reactive oxygen species (ROS) production via SDC-1. Therefore, it is possible that OPG binds with HS, and promotes SDC-1 co-receptor activation as described in their studies. Made by Biorender.

Osteoprotegerin (OPG) has seven structural domains, with domain 7 containing both a heparin-binding region and the free cysteine residue, which plays a crucial role in forming disulfide bonding to form a dimeric ligand. The OPG dimer can then associate with other molecules such as the receptor activator of nuclear factor-κB ligand (RANKL), TNF-related apoptosis-inducing ligand (TRAIL), and syndecan-1 (SDC-1) [1–4]. It is important to emphasize here that when OPG binds to RANKL and TRAIL, this effectively inhibits them from binding to each of their specific receptors RANK and TRAIL-R1-4, respectively. Therefore, OPG can function as an antagonist of RANKL- and TRAIL-induced signaling (Figure 1).

RANK is a transmembrane receptor with a cytoplasmic C-terminal domain that can bind to TNF receptor-associated factors (TRAFs, 2, 5, 6). RANKL-binding RANK activates TRAFs, leading to NF-kB and AP-1 activation. It has been reported that RANKL–RANK can increase VSMC calcification via NF-kB-mediated BMP4 induction [8]. OPG can function as an antagonist of RANKL-induced signaling, suggesting that in the context of RANKL–RANK-mediated VSMC calcification via TRAF–NF-κB–BMP4 signaling, OPG exerts an inhibitory effect on RANKL-induced NF-κB activation. Indeed, the depletion of OPG in ApoE^−/−^ mice exhibited the acceleration of not only vascular calcification, but also atherosclerosis formation [9]. Therefore, in this context, OPG functions as an anti-atherosclerotic factor probably by inhibiting vascular inflammation (Figure 1).

## The OPG–TRAIL interaction inhibits vascular apoptosis

TRAIL-R1 and R2 are type 1 transmembrane receptors containing an intracellular death domain (DD) but the association of TRAIL with TRAIL-R1 and 2 does not always induce apoptosis. In contrast, TRAIL–TRAIL-R1/2 can induce survival by activating PI3-K-Akt and ERK1/2 signaling. Particularly in vascular cells such as ECs and VSMCs, the induction of pro-survival pathway of PI3-K-Akt and ERK1/2 activation induced by TRAIL is more prominent than pro-apoptotic signaling after the exposure of these cells to ligand (Figure 1). For example, it has been reported that TRAIL inhibits interferon-γ, TNF-α, and IL-1β-induced VSMC apoptosis, and enhances VSMC migration and proliferation [10]. In endothelial cells (ECs), TRAIL activates PI3-K/Akt pathway and subsequently increases eNOS activity [11,12]. In fact, systemic administration of TRAIL inhibits atherosclerotic plaque formation, and genetic deletion of TRAIL accelerates it [13,14]. These data suggest that TRAIL possesses protective effects against vascular dysfunction and is atheroprotective. As stated above, since OPG inhibits TRAIL–TRAIL-R1/2 association, the detrimental effects of OPG on vascular function can also be explained by OPG-mediated inhibitory effects on TRAIL–TRAILR1/2 signaling.

## The OPG–SDC-1 interaction induces oxidative stress

The binding of OPG with syndecan family has been reported [15]. Syndecans are transmembrane proteoglycans (PGs) with a highly conserved C-terminal cytoplasmic domain, a single-pass transmembrane domain, and a large N-terminal extracellular domain. The extracellular domain of SDC-1 possesses five glycosaminoglycan (CAG) side chains consisting of heparan sulfate (HS) and chondroitin sulfate (CS). HS chains play a major role for SDC-1 to associate with heparin-binding growth factors and their receptors such as FGFs, VEGFs, Wnt, and HGF [15,16]. HS associates with both the growth factor and its receptor, stabilizes the complex. Therefore, SDC-1 can act as ‘co-receptor’, and plays a crucial role in growth factor activation (Figure 1). For example, 2-0 sulfate is critical for FGF2 and HS binding, leading to increase in FGF2 and its receptor binding, and promote FGFR-mediated signaling [17]. The activation of NADPH oxidase induced by growth factor receptor activation has been well established [18–22]. Therefore, it is reasonable to speculate that OPG–SDC-1 association induces NOX1/4 activation via various growth factor receptor activation.

## Conclusion

In the present study, Alves-Lopes et al. show that OPG can induce vascular dysfunction via inducing reactive oxygen species (ROS) production []; however, the atheroprotective role of OPG has been suggested in previous studies. At a glance, this seems to be contradictory, but it might be due to the different roles of inflammation and growth factor-induced ROS production during the process of particular cardiovascular events. It is well known that strong inflammatory events are instigated in significantly high cholesterol level-mediated atherosclerotic plaque formation in apoE^−/−^ mice. In this case, the anti-inflammatory effects of OPG on RANKL–RANK signaling may play a key role in inhibiting atherosclerotic plaque formation. In contrast, without significant hypercholesterolemia condition, anti-inflammatory effects of OPG may play only a minor role. Instead OPG-induced NOX1/2 activation via SDC-1 may become more critical in regulating vascular contraction and accelerating vascular dysfunction. The data of OPG effects on vascular dysfunction may suggest an importance of the stage and severity of the vascular dysfunction mediated by both inflammation and ROS production. Inflammatory factors may not equally contribute to the process of vascular dysfunction and consequent atherosclerotic plaque formation. Therefore, the effects of inflammation and ROS on the vasculature may be very different. Future studies may need to focus on the similarities and differences between responses to inflammation and ROS. To study the effects of OPG during the process of atherosclerosis may provide a good model.

## Data Availability

No new data are available in this manuscript.

## Open Access

Open access for this article was enabled by the participation of University of Texas MD Anderson Cancer Center in an all-inclusive *Read & Publish* pilot with Portland Press and the Biochemical Society under a transformative agreement with EBSCO.

## Abbreviations

eNOS: endothelial nitric oxide synthase
HS: heparan sulfate
NOX1/4: NADPH oxidase 1/4
OPG: osteoprotegerin
RANKL: receptor activator of nuclear factor-κB ligand
ROS: reactive oxygen species
SDC-1: syndecan-1
TNF: Tumor necrosis factor
TRAF: TNF receptor-associated factor
TRAIL: TNF-related apoptosis-inducing ligand
